# Implication of the Receptor Tyrosine Kinase AXL in Head and Neck Cancer Progression

**DOI:** 10.3390/ijms18010007

**Published:** 2016-12-22

**Authors:** Anne von Mässenhausen, Johannes Brägelmann, Hannah Billig, Britta Thewes, Angela Queisser, Wenzel Vogel, Glen Kristiansen, Andreas Schröck, Friedrich Bootz, Peter Brossart, Jutta Kirfel, Sven Perner

**Affiliations:** 1 Section of Prostate Cancer Research, University Hospital of Bonn, 53127 Bonn, Germany; anne.vonmaessenhausen@gmail.com (A.v.M.); j.braegelmann@gmx.de (J.B.); britta.thewes@gmail.com (B.T.); queisserangela@googlemail.com (A.Q.); 2 Institute of Pathology, University Hospital of Bonn, 53127 Bonn, Germany; Glen.Kristiansen@ukb.uni-bonn.de (G.K.); jutta.kirfel@ukb.uni-bonn.de (J.K.); 3 Center for Integrated Oncology Cologne/Bonn, University Hospital of Bonn, 53127 Bonn, Germany; Andreas.Schroeck@ukb.uni-bonn.de (A.S.); Friedrich.bootz@ukb.uni-bonn.de (F.B.); peter.brossart@ukb.uni-bonn.de (P.B.); 4 Department of Hematology/Oncology/Rheumatology, University Hospital of Bonn, 53127 Bonn, Germany; 5 Institute of Pathology, University Hospital of Luebeck, 23538 Luebeck, Germany; hannah.stefanie.billig@gmail.com (H.B.); wenzelvogel@googlemail.com (W.V.); 6 Leibniz Research Center Borstel, 23845 Borstel, Germany; 7 Department of Otorhinolaryngology/Head and Neck Surgery, University Hospital of Bonn, 53127 Bonn, Germany

**Keywords:** HNSCC, AXL, receptor tyrosine kinase, targeted therapy, BGB324, immunohistochemistry

## Abstract

Head and neck squamous cell carcinoma (HNSCC) remains a clinical challenge and identification of novel therapeutic targets is necessary. The receptor tyrosine kinase AXL has been implicated in several tumor entities and a selective AXL small molecule inhibitor (BGB324) is currently being tested in clinical trials for patients suffering from non-small cell lung cancer or acute myeloid leukemia. Our study investigates AXL expression during HNSCC progression and its use as a potential therapeutic target in HNSCC. AXL protein expression was determined in a HNSCC cohort (*n* = 364) using immunohistochemical staining. For functional validation, AXL was either overexpressed or inhibited with BGB324 in HNSCC cell lines to assess proliferation, migration and invasion. We found AXL protein expression increasing during tumor progression with highest expression levels in recurrent tumors. In HNSCC cell lines in vitro, AXL overexpression increased migration as well as invasion. Both properties could be reduced through treatment with BGB324. In contrast, proliferation was neither affected by AXL overexpression nor by inhibition with BGB324. Our patient-derived data and in vitro results show that, in HNSCC, AXL is important for the progression to more advanced tumor stages. Moreover, they suggest that AXL could be a target for precision medicine approaches in this dismal tumor entity.

## 1. Introduction

Although head and neck squamous cell carcinoma (HNSCC) is the sixth most common tumor entity worldwide, it remains a clinical challenge with five-year survival rates of around 50% depending on the stage at the time of diagnosis [1,2]. Currently the majority of advanced-stage patients are treated with unselective therapies such as cisplatin or docetaxel, which exhibit relevant toxicity. An increased understanding of the biological mechanisms leading to tumorigenesis and tumor progression is required to identify novel targets that may be exploited therapeutically in order to improve patient outcomes and reduce therapy-related toxicity. Similar to other cancer entities, receptor tyrosine kinases (RTKs) such as the insulin-like growth factor 1 receptor (IGF1R) [3], the MET proto-oncogene receptor tyrosine kinase (MET) [4], the vascular endothelial growth factor receptor (VEGFR) [5], the fibroblast growth factor receptor 1 (FGFR1) [6], and, especially, the epidermal growth factor receptor (EGFR) [7,8] have been explored as potential targets in HNSCC. However, so far, cetuximab is the only anti-EGFR treatment that showed beneficial effects for patients [9,10]. Moreover, treatment of patients with recurrent or metastatic HNSCC with the irreversible erythroblastosis oncogene B (erbB) family blocker afatinib improved progression-free survival [11].

The receptor tyrosine kinase (AXL) belongs to the family of TAM RTKs (TYRO3-AXL-MERTK) that also includes TYRO3 protein tyrosine kinase (TYRO3) and MER proto-oncogene, tyrosine kinase (MERTK). Binding of its ligand GAS6 leads to activation of several pathways such as the MAPK- and the PI3K/AKT signaling [12].

High expression of AXL is present in different solid and hematological tumors. In pancreatic cancer, AXL is expressed in 55% of patients and was shown to be involved in proliferation, anchorage independent growth, migration and invasion of cancer cells [13]. Similar results were found in hepatocellular carcinoma [14] and glioma [15]. In lung cancer, AXL plays an important role in the invasion of tumor cells [16], and its inhibition decreased tumor growth in xenograft models [17]. In HNSCC, AXL has been described to play a role in HNSCC primary tumors [18], but has so far not been studied in more advanced stages. To close this gap, we performed an immunohistochemistry (IHC) study on a large cohort of HNSCC patient samples including lymph node metastases and local recurrences.

Several small molecule inhibitors are being developed, but, currently, BGB324 (R248) is the only selective AXL inhibitor that is used in phase I clinical trials for patients suffering from non-small cell lung cancer or acute myeloid leukemia [19,20]. To assess whether AXL may represent a potential therapeutic target in HNSCC, we also investigated key oncogenic properties after AXL overexpression or inhibition in HNSCC cell line models.

## 2. Results

### 2.1. Analysis of AXL Expression in Patients with HNSCC

**Figure 1:**
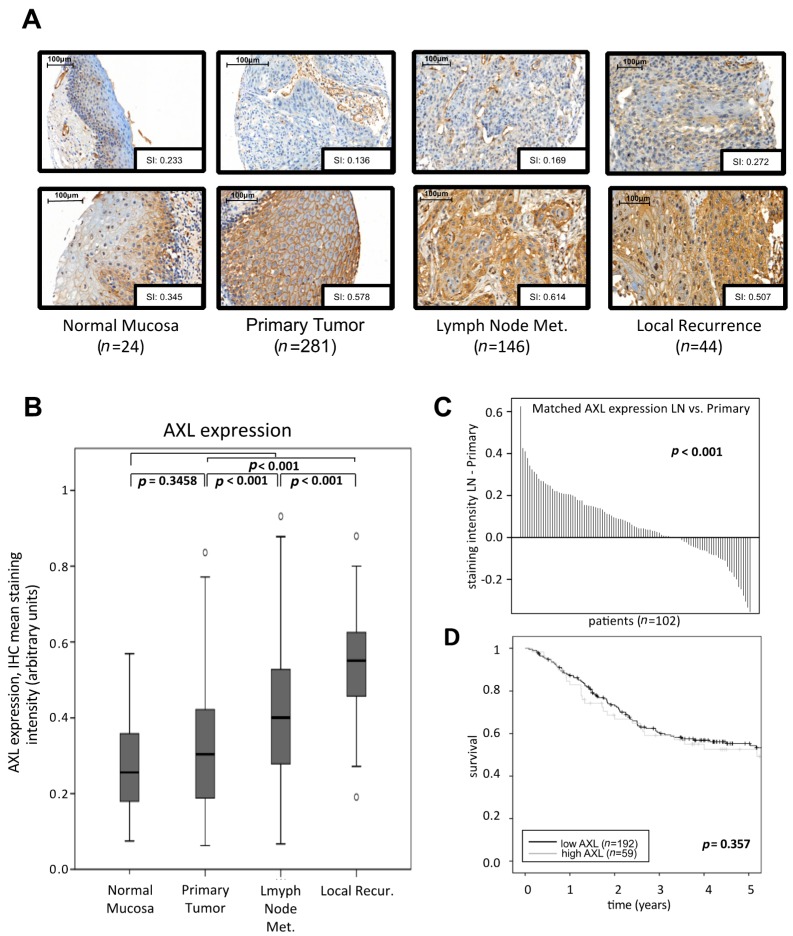
AXL expression in HNSCC patients. (**A**) IHC following staining for AXL. For each manifestation, representative cores with negative/weak (**top** row) or strong expression (**bottom** row) are shown. Expression is quantified by the mean membranous staining intensity (SI, arbitrary units) in the sample as calculated by the Definiens software; (**B**) summary of AXL protein expression by tumor stage; (**C**) difference in AXL expression between matched lymph node metastases and primary tumors was significantly higher in lymph node metastases compared to their matched primary tumor; and (**D**) Kaplan–Meier estimates for overall survival of patients in the highest quartile of AXL expression (**high** AXL) compared to all other patients (**low** AXL). Result of the univariate log-rank test is indicated. HNSCC, head and neck squamous cell carcinoma; IHC, immunohistochemistry; LN, lymph node metastases.

AXL protein expression was quantified by IHC in 495 tissue samples derived from 364 patients. Tissue samples included 24 cases of normal mucosa, 281 primary tumors, 146 lymph node metastases and 44 recurrences (Figure 1A). Clinical information was available for 321 (88.2%) of these patients, but AXL expression was not correlated with clinico-pathological parameters (Table 1).

Overall, we found a continuous increase of AXL expression during tumor progression with significantly higher levels in malignant specimens compared to normal mucosa (*p* < 0.001). A trend towards higher AXL protein expression was already present in normal mucosa vs. primary tumors, but did not reach significance (*p* = 0.35). In more advanced stages, the increases in AXL expression were significant: average expression was significantly higher in lymph node metastases compared to primary tumors (*p* < 0.001) and still higher in local recurrences compared to lymph node metastases (*p* < 0.001, Figure 1B), indicating the increasing importance of AXL during HNSCC tumor progression. Due to the large variation in expression values among patients of the same stage, we also compared the expression in matched primary tumors and lymph node metastases from patients for which both tissue types were available (*n* = 102). This paired analysis confirmed the increase seen in the unmatched analysis (*p* < 0.001, Figure 1C). Matched samples of primary tumors and local recurrences were only available for ten patients, but nevertheless showed a trend towards increased expression in local recurrences (*p* = 0.064). In a univariate survival analysis, AXL was no prognostic marker (five-year survival rate 53%, AXL high and 49% AXL low, *p* = 0.249) (Figure 1C). Similarly, a Cox regression model showed no survival difference after adjustment for age, tumor stage, human papillomavirus (HPV), alcohol abuse and tobacco consumption (*p* = 0.928, hazard ratio (HR) = 1.022, 95% CI 0.638–1.639, Table S1).

### 2.2. Effect of AXL Overexpression

**Figure 2:**
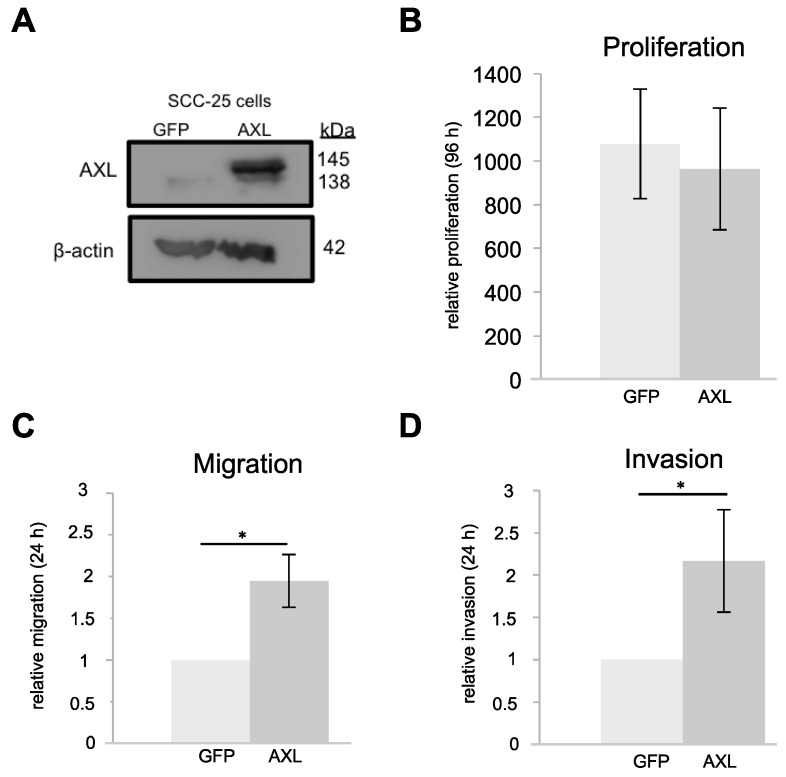
AXL overexpression in SCC-25 cells. (**A**) AXL overexpression in SCC-25 cells compared to GFP control cells. In the overexpression cells, the double band indicates expression of both endogenous AXL and GFP-tagged AXL; (**B**) relative proliferation of AXL overexpression and GFP control cells (*n* = 3); (**C**) relative migration of AXL overexpression and GFP control cells (*n* = 3); and (**D**) relative invasion of AXL overexpression and GFP control cells (*n* = 3). (**B**–**D**) two-tailed paired *t*-test, * *p* < 0.05). GFP, green fluorescent protein.

To investigate the function of AXL in HNSCC progression and to analyze its impact on different tumorigenic properties, we overexpressed GFP tagged AXL in SCC-25 cells, which have only little endogenous AXL expression (Figure 2A). Compared to cells with a vector expressing GFP alone, overexpression of AXL in SCC-25 cells had no effect on proliferation after 96 h (Figure 2B) but led to a two-fold increase of migration (Figure 2C, *p* < 0.05) as well as invasion after 24 h (Figure 2D, *p* < 0.05).

### 2.3. Effect of AXL Inhibition

**Figure 3:**
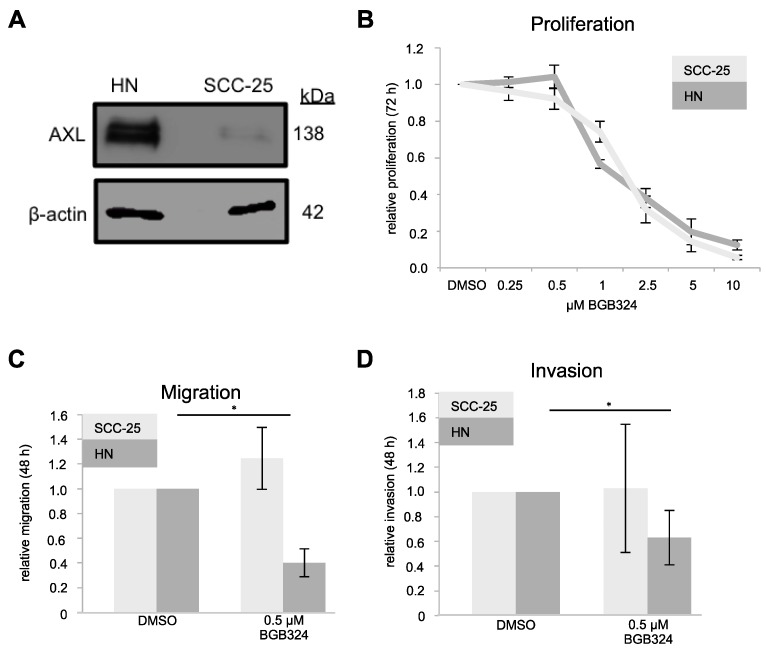
AXL inhibition with BGB324. (**A**) AXL expression in HN and SCC-25 cells; (**B**) relative proliferation of AXL high HN cells and AXL low SCC-25 cells after treatment with different amounts of BGB324 (*n* = 3, each in triplicates); (**C**) relative migration of AXL high HN cells and AXL low SCC-25 cells after pre-treatment with 0.5 µM BGB324 for 24 h (*n* = 4); and (**D**) relative invasion of AXL high HN cells and AXL low SCC-25 cells after pre-treatment with 0.5 µM BGB324 for 24 h (*n* = 4). (**C**,**D**) two-tailed paired *t*-test, *n* = 3, * *p* < 0.05). DMSO, Dimethyl sulfoxide.

To investigate AXL as a potential therapeutic target in HNSCC, we next analyzed the effects of AXL inhibition using the AXL selective small molecule inhibitor BGB324. To this end, SCC-25 cells derived from a primary tongue cancer [21] with low endogenous AXL protein expression and HN cells derived from a lymph node metastasis [22] with high endogenous AXL protein expression (Figure 3A) were treated with BGB324. Compared to Dimethyl sulfoxide (DMSO) treated controls, BGB324 led to reduced cell viability in both cell lines after 72 h without significant difference in cell viability between the two cell lines, indicating that proliferation was not primarily dependent on the level of AXL expression (Figure 3B).

Furthermore, we investigated the effect of AXL inhibition on cell motility. Pre-treatment of HN and SCC-25 cells with 0.5 µM BGB324 for 24 h led to 50% decreased migration and invasion in AXL high HN cells, whereas both properties were not affected in AXL low SCC-25 cells (*p* < 0.05, Figure 3C,D).

## 3. Discussion

Although HNSCC is a frequent tumor entity, its treatment remains a clinical challenge. Overexpression of the RTK AXL has been implicated in the tumorigenesis of several tumor entities such as pancreatic [13], liver [14], and breast cancer [23], glioma [15], and primary HNSCC [18].

In this study, we investigated for the first time AXL protein expression not only in primary tumors of the head and neck but also in lymph node metastases and recurrences. Similar to prior studies [18,24], we found lowest levels of AXL expression in normal mucosa and higher levels in malignant specimens. Interestingly, even normal tissues showed varying degrees of AXL expression with a gradient from basal to superficial layers, whereas tumors showed more homogeneous patterns, which may account for the lack of significant difference between normal mucosa and primary tumors. Even though the increase from normal to primary tumor tissue was non-significant, AXL expression further increased significantly in lymph node metastases and local recurrences, extending previous studies regarding HNSCC tumor progression. Due to the nature of our cohort, we were, moreover, able to confirm the increased AXL expression in matched primary tumor and lymph node metastases specimens from the same patient. Interestingly, SCC-25 cells (derived from a primary tongue cancer [21]) express low endogenous AXL protein levels and HN cells (derived from a lymph node metastasis [22]) have high AXL protein expression, which reflects the pattern seen in patients. The IHC findings thus indicate that AXL gains importance during tumor progression and should also be evaluated as a therapeutic target in more advanced stages.

In contrast to other studies [18,24], AXL expression was not associated with worse patient outcome in the present cohort. Apart from methodological aspects (Giles et al. [24] assessed AXL mRNA and Brand et al. [18] assessed AXL protein expression using a conventional categorical rating), differences might be due to cohort compositions. Interestingly, the 300 HNSCC patients from The Cancer Genome Atlas (TCGA) have a much lower long-term overall survival (10-year survival rate, 10% AXL high and 30% AXL low) [24] compared to our cohort (10-year survival rate, 53% AXL high and 42% AXL low). This indicates systematic differences in the patient populations under study and potential confounding by geographic distribution, ethnic background or therapeutic regimens. Brand et al. found a significantly decreased progression-free survival with high AXL expression in a smaller cohort of 63 patients [18]. However, direct comparison with the study of Giles et al. [24] or the present one is hampered by the lack of adjusted analyses and of information regarding overall survival as well as potential systematic differences as mentioned above. Similar discrepancies regarding associations of AXL expression with clinico-pathological parameters and survival were also found in other tumor entities such as non-small cell lung cancer [16,25].

When analyzing the effect of AXL on tumorigenic properties in HNSCC cell lines, AXL did not appear to relevantly influence cell viability, as indicated by unchanged proliferation in cells overexpressing AXL. Similarly, in glioma and melanoma, AXL overexpression did not alter proliferation [15,26]. Treatment with the small molecular inhibitor BGB324 decreased proliferation only at increased concentrations with comparable effects in HN cells with high AXL expression and in SCC-25 cells that display considerably lower AXL protein levels. It therefore cannot be excluded that this is not primarily an AXL-specific effect, but reduced viability may also be due to off-target effects on other tyrosine kinases, as described previously for BGB324 at higher doses [27]. In a distinct set of HNSCC cells, Brand et al. [18] also showed that treatment with BGB324 led to decreased proliferation in AXL positive, but not in AXL negative cell lines, with comparable IC_50_ values to HN and SCC-25. However, the observed IC_50_ values in AXL positive cells were independent of the respective AXL protein expression levels [18], which may be reflected in the comparable effects of BGB324 in our AXL high HN and AXL low SCC-25 cells. The validity of presence vs. absence of AXL expression as a predictive marker regarding AXL-targeted treatment strategies therefore needs to be confirmed in further studies, ideally involving in vivo and ultimately clinical trials.

Apart from proliferation, migration and invasion are crucial properties for progression to more advanced tumor stages and for metastatic spread. These cell traits were relevantly altered by interfering with the AXL signaling axis. Overexpression of AXL in SCC-25 cells, e.g., led to a significantly increased migration and invasion, which corresponded well to the observed increase of AXL expression in lymph node metastases and local recurrences in the patient samples. More importantly, migration and invasion of head and neck cancer cells could be reduced by anti-AXL treatment with BGB324. As mentioned above, BGB324 may exhibit off-target effects at high doses [27]. For migration and invasion experiments, a concentration was therefore chosen that did not impact viability in either HN or SCC-25 cells. Whereas reduction in viability did not depend on the protein expression level, significant reductions in migration and invasion were only observed in HN cells, which show a high endogenous AXL expression, but not in SCC-25 cells. Together with the effects of AXL-overexpression in SCC-25 cells, these differential results suggest that AXL can be a relevant player in HNSCC cell motility, which is in line with our observation that AXL protein expression is increased in more advanced tumor stages and lymph node metastases compared to matched primary tumors, and validates findings in other tumor entities such as neuroblastoma [28] und ovarian cancer [29].

Taken together, our patient-derived data and in vitro results suggest that, in head and neck cancer, AXL is important for the progression to more advanced tumor stages and is functionally involved in HNSCC cell motility. Given the modest effects on cell viability in this entity demonstrated by us and others [18,24], an anti-proliferative treatment based solely on AXL-inhibition will probably not suffice. However, the combination of an AXL-targeting treatment with the existing standards of care, such as radio-chemotherapy, should be further explored in patients that are AXL-positive or present with lymph node metastases to prevent further disease progression and to offer novel therapeutic options.

## 4. Materials and Methods

### 4.1. Immunohistochemical Staining

AXL protein expression was evaluated by immunohistochemical staining of tissue microarrays (TMA) from a cohort of HNSCC patients (*n* = 364). Patients were treated at the University Hospital Bonn, Bonn, Germany between 1997 and 2011 and were clinically annotated as described previously (Bonn HNSCC cohort) [30]. The study was approved by the institutional review board of the University of Bonn (#148/11; 04-07-2011). Immunohistochemical staining was conducted as described previously [31].

Antibodies (used in 1:500 dilution) were the following: AXL (R&D Systems, Minneapolis, MN, USA, AF154) and Rabbit Anti-Goat IgG (H+L, Jackson ImmunoResearch, West Grove, PA, USA). AXL protein expression on scanned slides (Pannoramic Desk, 3DHistech, Budapest, Hungary) was quantified with Definiens Tissue Studio 2.1 software (Definiens Inc., Munich, Germany) as described previously [32]. To exclude stroma, tumor areas in each sample were marked manually blinded to types of samples (e.g., primary vs. recurrence) and patient outcomes. Average sample staining intensity (SI) in these regions of interest was measured as a continuous value (range 0.063–0.932, arbitrary units) by the Definiens software (Definiens Inc., Munich, Germany), with higher values indicating stronger staining [32].

### 4.2. Cell Lines

SCC-25 cells were obtained from the American Type Culture Collection (ATCC), (Manassas, VA, USA) and HN cells were purchased from the Leibnitz Institute DSMZ (Deutsche Sammlung von Mikroorganismen und Zellkulturen), Braunschweig, Germany. Authenticity of cells was verified by SNP-Profiling (Multiplexion, Heidelberg, Germany) in January 2014. SCC-25 cells were maintained in Dulbecco’s Modified Eagle Medium/Nutrient Mixture F-12 (DMEM/F-12) (Gibco^®^ Life Technologies, Darmstadt, Germany) supplemented with 10% fetal bovine serum (FBS) (Biochrom, Berlin, Germany), 2 mM l-Glutamine (Gibco^®^ Life Technologies), 400 ng/mL hydrocortisone (Sigma-Aldrich, Munich, Germany) and 1% Penicillin Streptomycin (Gibco^®^ Life Technologies). HN were cultured according to the manufacturer’s instructions (DMEM with l-Glutamine (Gibco^®^ Life Technologies)) with 10% FBS and 1% Penicillin Streptomycin).

### 4.3. Generation of AXL Overexpressing Cells

pDONR223-AXL was obtained from Addgene, Cambridge, MA, USA (Addgene plasmid # 23945) [33]. AXL cDNA was cloned into the pLenti-C-mGFP (OriGene, Rockville, MD, USA) and SCC-25 cells were transduced with this vector or empty vector as control using lentiviral transduction. Fluorescence-activated cell sorting (FACS) of GFP+ cells (FACSAriaIII, BD Biosciences, Heidelberg, Germany) was used to select positive cells. Overexpression was subsequently estimated via Western blot.

### 4.4. Proliferation

Thiazolyl Blue Tetrazolium Bromide (MTT) was used to evaluate viability as described previously [34,35]. In addition, 1500 SCC-25 cells overexpressing AXL or respective control cells were plated in 100 µL medium in 96-Well plates (Corning, Corning, NY, USA). Furthermore, 500 µg/mL of MTT (Sigma-Aldrich) solution was prepared in phosphate buffered saline (PBS) (Gibco^®^ Life Technologies) and was added to the sample wells at indicated times. Four hours later, solubilization buffer was added and absorbance at 595 nm was measured after 24 h. For inhibition experiments, 2500 HN or SCC-25 cells were plated in 50 µL medium before adding different amounts of BGB324 (BerGenBio, Bergen, Norway) in 50 µL medium the next day. MTT was added after different time points as described above. Each experiment was performed in triplicate per experiment and concentration. Experiments were repeated at least three times.

### 4.5. Migration and Invasion

For migration and invasion assays, 1 × 10^5^ HN or SCC-25 cells were seeded in 2% FBS containing media in the upper chambers of migration (VWR, Darmstadt, Germany) and invasion (VWR) inserts. The lower chamber was filled with medium containing 10% FBS. After 24 or 48 h, migrated/invaded cells were fixed with 4% paraformaldehyde (Merck, Darmstadt, Germany), stained with hemalaun (Waldeck, Münster, Germany) and washed with water. Subsequently, 5–10 representative areas of the membrane were counted. For inhibition experiments, cells were pre-treated with 0.5 µM BGB324 or the respective amount of DMSO for 24 h before performing migration and invasion experiments. Experiments were repeated three times for AXL overexpression and four times for BGB324 treatment.

### 4.6. Western Blot

For protein extraction, cells were lysed for 1 h on ice with radioimmunoprecipitation assay (RIPA) buffer before centrifuging at 13,000× *g*, 4 °C for 30 min. Protein concentration was measured using Pierce™ BCA Protein Assay Kit (Gibco^®^ Life Technologies). Blotting was done on polyvinylidene fluoride (PVDF) membranes, blocked with 5% bovine serum albumin (BSA) or milk in TBS/0.05% Tween prior to overnight incubation with primary antibodies. Primary antibodies: AXL (#8661), (Cell Signaling, Danvers, MA, USA), β-actin (A2228) (Sigma-Aldrich). After washing with TBS/0.05% Tween, followed by incubation with HRP-conjugated antibodies against rabbit (#7074) or mouse Ig (#7076) (Cell Signaling) in 5% milk TBS/0.05% Tween for one h at room temperature, developing was carried out using enhanced chemiluminescence (ECL) Western Blotting Reagent (GE Healthcare, Munich, Germany).

### 4.7. Statistics

Differences of continuous variables between two groups were tested at a two-sided significance level of 0.05 with Students *t*-test for normally distributed data or non-parametric Mann–Whitney-U test otherwise and the Kruskal–Wallis-H test for more than two nominal categories. Survival was analyzed using Kaplan–Meier estimators with log-rank test and Cox regression models with adjustment for clinical covariables [36,37]. All statistical analyses were performed using IBM SPSS Statistics 22 (IBM, Ehningen, Germany).

## Figures and Tables

**Table 1:** Clinico-pathological features of the cohort used for AXL expression analyses. Bonn HNSCC Cohort (*n* = 364 patients).

Clinical Parameter	Number	Median AXL Expression	Statistics
**Available Tissues**			
Normal Mucosa	24 (13 ^a^)	0.256	*p* < 0.0001 ^b^ (normal mucosa vs. primary tumor, lymph node metastasis and recurrence)
Primary Tumor	281 (17 ^a^)	0.304	
Lymph node metastasis	146 (14 ^a^)	0.401	
Recurrence	44 (5 ^a^)	0.551	
**Patients with Clinical Data (*n* = 321)**			
**Gender**			
Male	240 (74.8%)	0.304	*p* = 0.108 ^b^
Female	81 (25.2%)	0.250	
**Age (Years, SD)**	61.7 (11.7)		
**Age**			
<54	86 (26.8)	0.305	*p* = 0.635 ^c^
54–62	84 (26.2)	0.272	
62–70	80 (24.9)	0.342	
>70	71 (22.1)	0.255	
**Anatomic Localization of Primary Tumor**			
Oral Cavity	80 (24.9%)	0.295	*p* = 0.229 ^c^
Oropharynx	117 (36.5%)	0.246	
Hypopharynx/Larynx	116 (36.1%)	0.317	
Unknown	8 (2.5%)		
**Tobacco**			
Never-Smoker	27 (8.4%)	0.290	*p* = 0.13 ^b^
Ever-Smoker	223 (69.5%)	0.351	
Unknown	71 (22.1%)		
**Alcohol**			
Non-drinker	89 (27.7%)	0.312	*p* = 0.112 ^c^
Occasional	58 (18.1%)	0.351	
Medium-Heavy	85 (26.5%)	0.244	
Unknown	89 (27.7%)		
**HPV Status**			
Positive	30 (9.3%)	0.295	*p* = 0.429 ^b^
Negative	291 (90.7%)	0.338	
**T-Stage of Primary**			
T1	76 (23.7%)	0.299	*p* = 0.324 ^c^
T2	119 (37.1%)	0.328	
T3	72 (22.4%)	0.274	
T4	50 (15.6%)	0.242	
Unknown	4 (1.2%)		
**N Stage of Primary**			
N0	137 (42.7%)	0.288	*p* = 0.495 ^c^
N1	48 (15.0%)	0.325	
N2	124 (38.6%)	0.260	
N3	5 (1.5%)	0.288	
Unknown	7 (2.2%)		
**M Stage of Primary**			
M0	305 (95.0%)	0.290	*p* = 0.32 ^b^
M1	14 (4.4%)	0.227	
Unknown	2 (0.6%)		

^a^ Number of tissue samples of patients without clinical information; for a number of patients, tissue for more than one entity (e.g., normal and primary tumor) was available; ^b^ Mann–Whitney-U-test; ^c^ Kruskal–Wallis-H-test; HNSCC, head and neck squamous cell carcinoma; SD, standard deviation; HPV, human papillomavirus.

## Supplementary Materials

Supplementary materials can be found at www.mdpi.com/1422-0067/18/1/7/s1.

## Abbreviations

AXL	AXL receptor tyrosine kinase
EGFR	Epidermal growth factor receptor
HNSCC	Head and neck squamous cell carcinoma
IHC	Immunohistochemistry
RTK	Receptor tyrosine kinase
SI	Average sample staining intensity
TMA	Tissue microarray
