# Supplementary material for: Implication of the Receptor Tyrosine Kinase AXL in Head and Neck Cancer Progression
Source: Int J Mol Sci. 2016 Dec 22;18(1):7. doi: 10.3390/ijms18010007 (PMC5297642; doi:10.3390/ijms18010007)
Supplement: Supplementary file 1 [file ijms-18-00007-s001.pdf]

Int. J. Mol. Sci. **2016**, *18*, 7; doi:10.3390/ijms18010007

Supplementary Materials: Implication of the Receptor Tyrosine Kinase AXL in Head and Neck Cancer Progression

Anne von Mässenhausen, Johannes Brägelmann, Hannah Billig, Britta Thewes, Angela Queisser, Wenzel Vogel, Glen Kristiansen, Andreas Schröck, Friedrich Bootz, Peter Brossart, Jutta Kirfel and Sven Perner

Table S1. Cox Model for the Bonn HNSCC cohort.

Cox Model for Bonn HNSCC Cohort (Overall Model *p*-Value 0.006)				
	**Covariable**	**Hazard ratio**	**95 % Conf. Interval**	***p*-Value**
High AXL	[true]	1.082	0.672–1.743	0.745
Age	[years]	1.012	0.986–1.038	0.383
UICC Stage	[II vs. I]	1.965	0.745–5.183	0.172
	[III vs. I]	2.657	1.025–6.886	**0.044**
	[IV vs. I]	3.267	1.426–7.484	**0.005**
HPV status	[positive]	0.339	0.121–0.950	**0.040**
Tobacco	[never-smoker vs. ever smoker]	1.044	0.487–2.237	0.911
Alcohol	[occasional vs. Non-drinker]	0.618	0.305–1.251	0.181
	[medium-heavy vs. Non-drinker]	1.512	0.860–2.658	0.151

Multivariable Cox models evaluating effects of AXL expression for patients with AXL expression above the median (High AXL) adjusted for clinical parameters known to be associated with prognosis in HNSCC.
